# Milk-borne small extracellular vesicles: kinetics and mechanisms of transport, distribution, and elimination

**DOI:** 10.20517/evcna.2023.25

**Published:** 2023-07-12

**Authors:** Alice Ngu, Javaria Munir, Janos Zempleni

**Affiliations:** Department of Nutrition and Health Sciences, University of Nebraska-Lincoln, Lincoln, NE 68583-0806, USA.

**Keywords:** Drug delivery, feces, lysosomes, macrophages, urine

## Abstract

Small extracellular vesicles (sEVs) in milk have the qualities desired for delivering therapeutics to diseased tissues. The production of bovine milk sEVs is scalable (10^21^ annually per cow), and they resist degradation in the gastrointestinal tract. Most cells studied to date internalize milk sEVs by a saturable process that follows Michaelis-Menten kinetics. The bioavailability of oral milk sEVs is approximately 50%. In addition to crossing the intestinal mucosa, milk sEVs also cross barriers such as the placenta and blood-brain barrier, thereby enabling the delivery of therapeutics to hard-to-reach tissues. In time course studies, levels of milk sEVs peaked in the intestinal mucosa, plasma, and urine approximately 6 h and returned to baseline 24 h after oral gavage in mice. In tissues, milk sEV levels peaked 12 h after gavage. Milk sEVs appear to be biologically safe. No cytokine storm was observed when milk sEVs were added to cultures of human peripheral blood mononuclear cells or administered orally to rats. Liver and kidney function and erythropoiesis were not impaired when milk sEVs were administered to rats by oral gavage for up to 15 days. Protocols for loading milk sEVs with therapeutic cargo are available. Currently, the use of milk sEVs (and other nanoparticles) in the delivery of therapeutics is limited by their rapid elimination through internalization by macrophages and lysosomal degradation in target cells. This mini review discusses the current knowledge base of sEV tissue distribution, excretion in feces and urine, internalization by macrophages, and degradation in lysosomes.

## INTRODUCTION

Small extracellular vesicles (sEVs) in milk have garnered attention for delivering therapeutics to diseased tissues. Most papers referenced in this mini review used exosome-rich preparations of sEVs, as evidenced by sizes that averaged 100 nm, the detection of exosome marker proteins, and the low abundance of markers of microsomes and nanoparticles such as lipoproteins, fat globules, and casein micelles^[1-3]^. It is safe to suggest that all preparations were contaminated with sEVs other than exosomes to some extent^[4]^. The National Nanotechnology Initiative defines nanoparticles as particles no larger than 100 nm in diameter, which might be relevant when using milk sEVs (“exosomes”) in nanomedicine^[5]^. Milk sEVs hold great promise in nanomedicine based on the following qualities. The production of milk sEVs is scalable, e.g., bovine milk contains approximately 10^14^ sEVs per mL, and cows produce approximately 10,800 kg of milk annually^[6,7]^. Milk sEVs protect their cargo against degradation in the gastrointestinal tract and industrial processing. Cells internalize milk sEVs by endocytosis, which confers a pathway for the delivery of therapeutic cargo that otherwise might not easily cross cell membranes^[8-11]^. Protocols have been reported for loading sEVs, including those from milk, with therapeutic cargos, including drugs, small RNAs, and proteins^[12-16]^. Milk sEVs are absorbed following oral administration and accumulate primarily in the intestinal mucosa, brain, spleen, and liver^[13,17,18]^. Milk sEVs do not elicit detectable immune reactions in humans and mice and do not compromise erythropoiesis, liver and kidney function, food and water intake, and body weight gain in rats and mice^[18-20]^. Milk sEVs cross barriers such as the placenta and blood-brain barrier and elicit changes in gene expression^[21,22]^.

The qualities discussed above have triggered an interest in utilizing milk sEVs for delivering therapeutics and diagnostics to hard-to-reach diseased tissues and tumors. Initial studies yielded promising results. For example, when bovine milk sEVs were loaded with lung cancer KRAS^G12S^-specific siRNA and delivered to athymic nude mice by intravenous injection, the volume of A549 lung cancer xenografts was 54% smaller compared to the vehicle control^[14]^. Results were similar when drug-loaded bovine milk sEVs were administered orally. For example, when bovine milk sEVs were loaded with paclitaxel and administered to female athymic nude mice by oral gavage, the growth of A549 lung cancer xenografts was decreased by > 50% and > 60% compared to free paclitaxel and untreated controls, respectively^[12]^. The pharmaceutical industry has recognized the potential for using bovine milk sEVs to deliver anti-cancer drugs, as evidenced by a $1 billion licensing agreement between Roche, Inc. and PureTech Health, Inc.^[23]^.

There are limitations to using milk sEVs in drug delivery, including their rapid elimination from body fluids. This mini review discusses the current knowledge base on milk sEV elimination in feces and urine, elimination by macrophages, and degradation in lysosomes. The discussion is framed in the context of milk sEV distribution among tissues and cell compartments. While recent reviews have discussed the roles of milk sEVs in nutrition and drug delivery, this mini review is unique because of its focus on pharmacokinetics, including the kinetics of uptake and elimination^[24-28]^.

## KINETICS OF MILK SEV INTERNALIZATION IN CELL CULTURES

Most cells studied to date internalize milk sEVs through a saturable process that follows Michaelis-Menten kinetics. Maximal rate of transport (V_max_) and Michaelis constant (K_m_) have been reported for bovine milk sEVs internalization in mouse brain endothelial bEnd.3 cells, mouse BV2 microglia, human colon carcinoma Caco-2 cells, rat small intestinal IEC-6 cells, and human umbilical cord HUVEC cells are summarized in Table 1^[17,22,29]^. Non-transformed human intestinal epithelial crypt-like HIEC cells appear to internalize human milk sEVs, but transport kinetics were not assessed^[30]^. Many of these studies used lipophilic dyes for labeling sEVs. Lipophilic dyes may detach from sEV membranes and transfer to other lipophilic particles and compounds, which is a potential limitation of these reports^[31]^. Studies in cell cultures cannot replace bioavailability studies in humans and animal models, but they can provide important mechanistic insights into sEV internalization and transport across layers of recipient cells[32]. For example, Z-stack microscopy was used to demonstrate that bEnd.3 cells internalized sEVs using labeled RNA as the tracer, as opposed to the sEVs adsorbing to the cell surface[22]. Dual chamber systems were used to demonstrate that milk sEVs monolayers are transported across monolayers of bEnd.3 and Caco-2 cultures[17,22]. Reporter plasmid studies provided experimental evidence that human peripheral blood mononuclear cells internalized unlabeled milk sEVs[33]. It is unknown what constitutes a level of natural, unloaded milk sEVs that elicits a biological effect in recipient cells.

## TIME COURSES OF MILK SEVS IN FECES, BODY FLUIDS, AND TISSUES FOLLOWING ORAL ADMINISTRATION

Data are scarce regarding the fecal and urinary excretion of milk sEVs. When bovine milk sEVs were labeled with a carbonyl-reactive fluorescent dye, HiLyte-750^™^, and administered by oral gavage, approximately 50% of the dose was recovered in feces, and excretion was complete within 24 h^[34]^. Incubation of HiLyte-labeled milk sEVs in artificial intestinal fluid provided experimental evidence that the label was not released and, therefore, sEVs (as opposed to free dye) were traced. In time course studies, levels of milk sEVs peaked in the intestinal mucosa, plasma, and urine approximately 6 h and returned to baseline 24 h after oral gavage in mice. In tissues, milk sEV levels peaked 12 h after gavage. It is possible that some of the fecal sEVs have gone through enterohepatic circulation, i.e., were excreted through bile into the gut after absorption, like the enterohepatic circulation of cholesterol and bile acids^[35]^. Similarly, it is possible that milk sEVs were released into the intestinal lumen by the shedding of intestinal cells. Approximately 2 × 10^8^ cells are shed from the small intestine per day in mice^[36]^.

The acidic environment in the stomach might cause changes in sEV morphology and partial degradation of the tetraspanins CD9 and CD81^[37]^. The implications for intestinal absorption of milk sEVs are unknown. Likewise, it is unknown whether changes in milk sEV size and morphology, caused by industrial processing, have an effect on the bioavailability and disposition^[38]^.

When bovine milk sEVs were loaded with fluorophore (IRDye)-labeled RNA and administered by oral gavage, strong fluorescence was observed in the kidney 6 h after administration in mice^[13]^. This is consistent with the time courses of urinary excretion of HiLyte-750^™^-labeled bovine milk sEVs in mice, which peaked 6 h after oral gavage^[34]^.

Dose-response time-course studies of the pharmacokinetics of human embryonic kidney Expi293F cell-derived sEVs in macaques (*Macaca nemestrina*) showed that the plasma half-life decreased from approximately 40 min for the low doses tested to 11 min for the highest dose tested^[39]^. The authors did not report the number of biological repeats, and it is unlikely that the pharmacokinetics are the same for kidney cell-derived sEVs and milk sEVs.

## ELIMINATION BY MACROPHAGES

**Figure 1.**
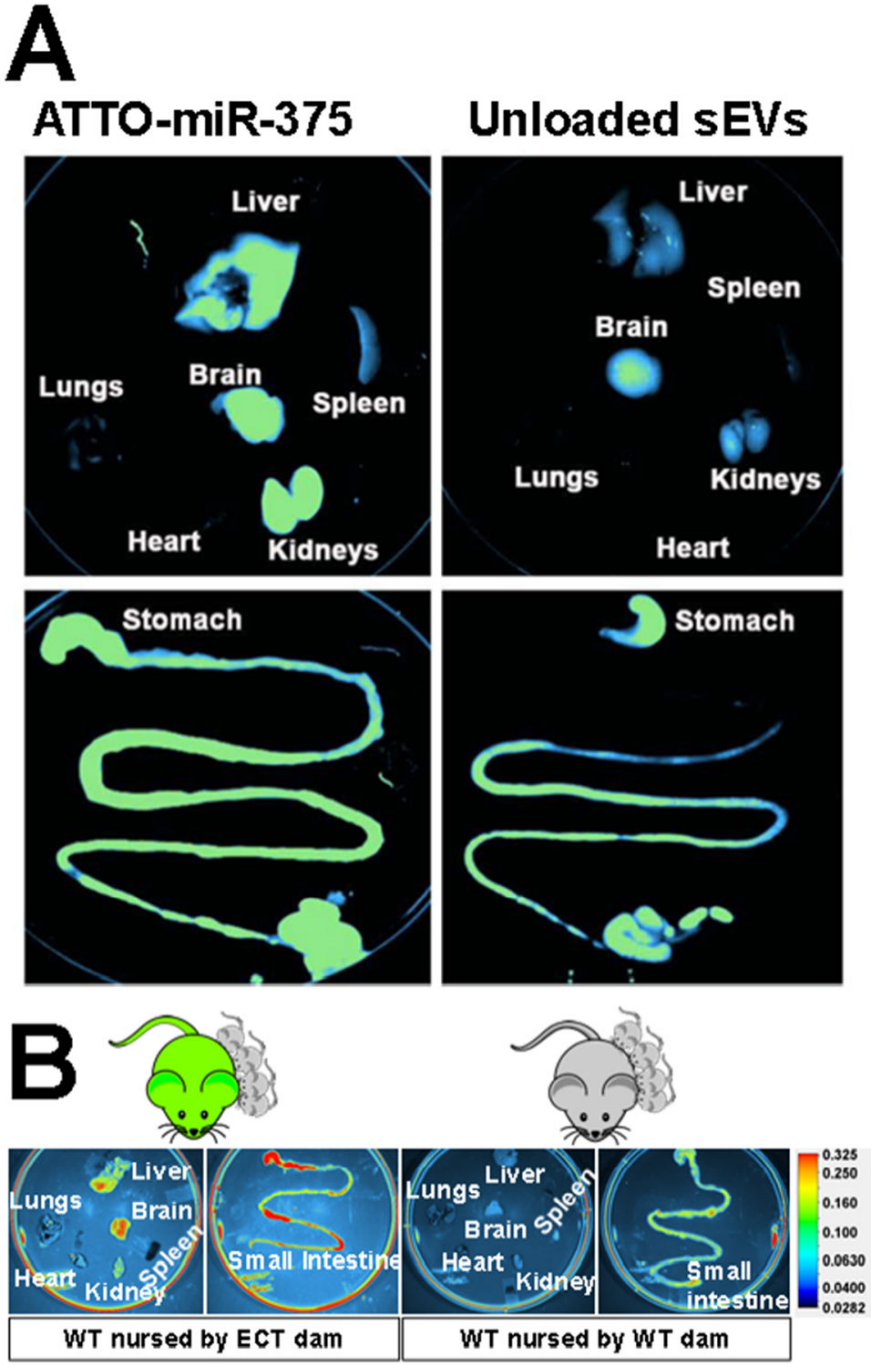
Tissue distribution of milk sEVs in mice. (A) Distribution of bovine milk sEVs loaded with fluorophore (ATTO)-labeled miR-375 in Balb/c mice. Modified from Manca *et al*. with permission from the original publisher, Nature Springer^[13]^. To view a copy of this license, visit http://creativecommons.org/licenses/by/4.0/; (B) Accumulation of enhanced green fluorescence protein (eGFP)-positive milk sEVs in peripheral tissues and the small intestinal mucosa in wild-type (WT) pups fostered to exosome and cargo tracking (ECT) dams and nursed for 17 days. ECT mice secrete sEVs labeled with an eGFP fusion protein in milk. WT pups fostered to WT dams served as controls. From Zhou *et al*. with permission from the original publisher, Frontiers Media SA^[22]^. To view a copy of this license, visit http://creativecommons.org/licenses/by/4.0/.

Macrophages play an important role in the clearance of foreign sEVs, including milk sEVs[11,40-42]. Milk sEV distribution studies provided circumstantial evidence for the role of macrophages in milk sEV elimination by demonstrating that bovine milk sEVs accumulated in large quantities in macrophage-rich tissues such as spleen and liver in addition to the brain [Figure 1A][13,18]. The liver was also a major accumulation site, in addition to intestinal mucosa and brain, when milk sEVs were obtained from the same species, i.e., when neonate mice were nursed by a dam that secreted milk sEVs endogenously labeled with a fluorescent fusion protein [Figure 1B][22]. The fusion protein studies provide compelling evidence that milk sEVs, not detached labels, were traced. Dual chamber protocols, Z-stack confocal microscopy, and serial two-photon tomography were used to demonstrate that milk sEVs cross the intestinal and blood-brain barriers and are internalized by target cells[17,22]. The uptake of bovine milk sEVs was formally assessed in murine bone marrow-derived macrophages and revealed that Class A scavenger receptor 1/2 is the primary transporter facilitating sEV internalization by macrophages[11]. Receptor knockout decreased sEV uptake by approximately 60%. Transporter capacity in macrophages exceeded capacities observed in intestinal, vascular, and brain endothelial cells[17,22,29]. When murine melanoma-derived sEVs were administered to macrophage-depleted mice, the mean residence time in plasma and clearance were 5.2-fold and 63-fold, greater and smaller, respectively, compared to control mice [Table 2][42]. It remains to be determined if the effects are similar for milk sEVs. Experimental evidence suggests that milk sEVs transport oligosaccharides into macrophages and modulate immunity and attenuate *Escherichia coli* infection in mice[43].

## DEGRADATION IN LYSOSOMES AND ESCAPE PATHWAYS

**Figure 2.**
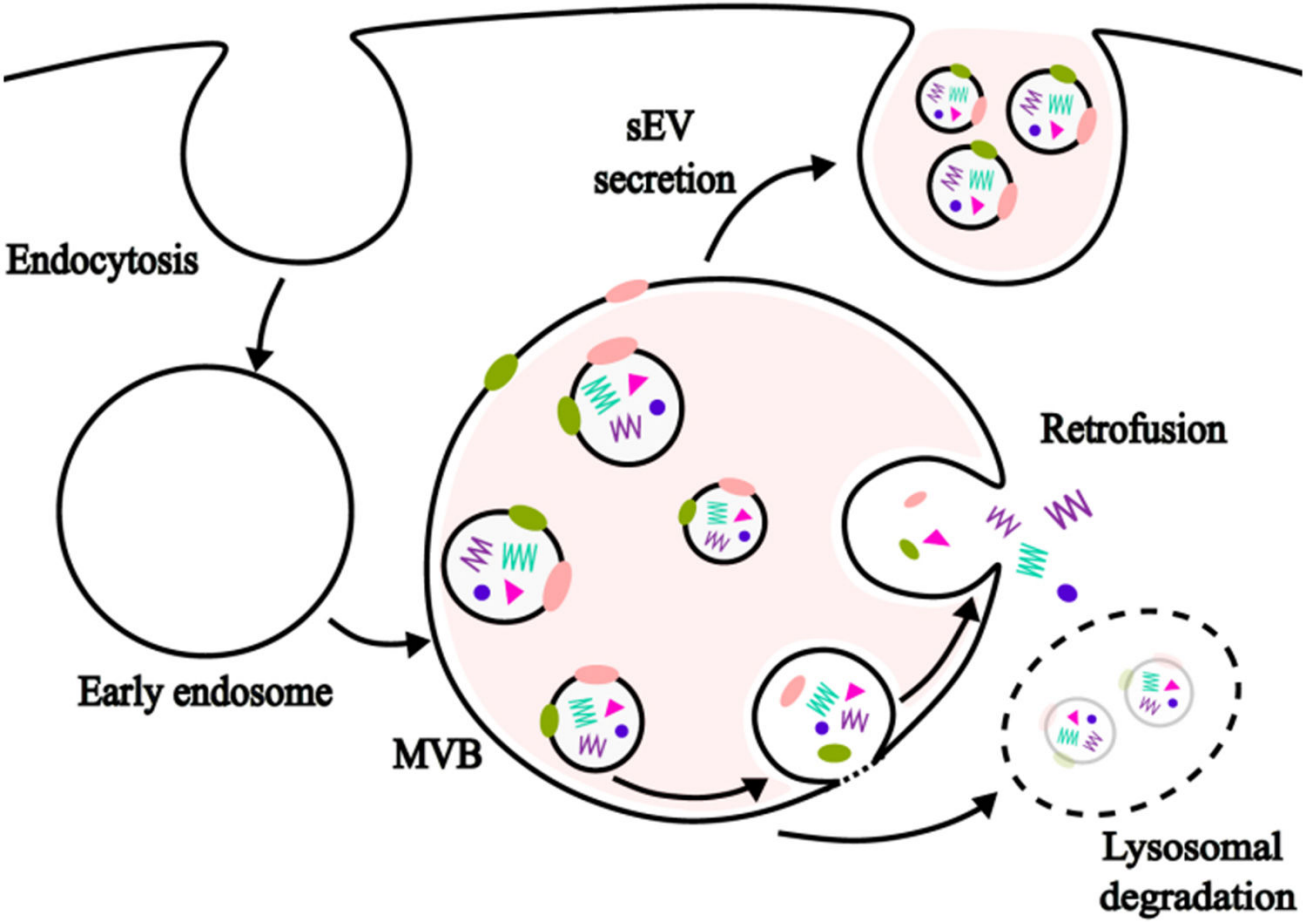
sEV uptake, degradation, and retrofusion. MVB: multivesicular body; sEV: small extracellular vesicle.

Intracellular bovine milk sEVs accumulated in cytoplasm and cytoplasmic organelles (presumably lysosomes and multivesicular body), whereas nuclear localization was not apparent in murine bone marrow-derived macrophages[11]. The biogenesis of sEVs occurs in the endosomal system, including multivesicular bodies[44]. sEVs and their cargos have three metabolic fates [Figure 2]: delivery to lysosomes for degradation, retrofusion to the limiting membrane of the multivesicular body and subsequent release of cargo into the cytoplasm, and fusion with the plasma membrane for subsequent release into the extracellular space[45,46]. A small fraction of sEVs escape lysosomal degradation, e.g., only approximately 17% of sEVs in human melanoma MelJuSo cells fused back to the limiting membrane of the multivesicular body[46]. No reports are available regarding the percentage of milk sEVs released into the extracellular space by fusion with the plasma membrane.

## CONCLUSION

Milk sEVs have properties conducive to their use in drug delivery, including, but not limited to, the transfer across the blood-brain barrier. A substantial fraction of milk sEVs is cleared by macrophages and presumably degraded in lysosomes in target cells. It is desirable to devise technologies that lowermacrophage uptake and lysosomal degradation of milk sEVs to realize their full potential in the delivery of therapeutic agents.

## Figures and Tables

**Table I.** Kinetics of milk sEV internalization in mammalian cell lines (from [2, 13, 19]).

Cell	V_max_	Unit	Km	Unit
bEnd.3	0.77 ± 0.18	ng sEV protein x 19,000 cells^−1^ x 45 min^−1^	1.8 ± 2.0	10^11^ sEV protein x mL media^−1^
BV2	0.66 ± 0.14	ng sEV protein x 19,000 cells^−1^ x 45 min^−1^	1.9 ± 1.9	10^11^ sEV protein x mL media^−1^
Caco-2	0.083 ± 0.057	ng sEV protein x 81,750 cells^−1^ x h^−1^	55.5 ± 48.6	μg sEV protein x 200μL media^−1^
IEC-6	0.14 ± 0.01	ng sEV protein x 36,375 cells^−1^ x h^−1^	152 ± 39.5	μg sEV protein x 200μL media^−1^
HUVEC	0.057 ± 0.004	ng sEV protein x 40,000 cells^−1^ x h^−1^	17.97 ± 3.84	μg sEV protein x 200μL media^−1^

Abbreviations: Km, Michaelis constant; sEV, small extracellular vesicle; V_max_; maximal velocity (transport rate).

**Table II.** Elimination kinetics of sEVs from murine melanoma B16BL6 cells in macrophage-depleted and control Balb/c mice. Macrophages were depleted by treating mice with clodronate.

Mice	sEV dose (μg)	MRT (h)	CL (mL/h)
Depleted	5.0	3.6±0.7*	0.65±0.16*
Control	5.0	0.69±0.09	41.2±6.8

Table adapted from T. Imai et al. [23].

* *P* <0.05 versus control (*n* = 4).

CL, clearance (plasma); h, hour; MRT, mean residence time; sEV, small extracellular vesicle.
